# *Pistacia atlantica* Resin Has a Dose-Dependent Effect on Angiogenesis and Skin Burn Wound Healing in Rat

**DOI:** 10.1155/2013/893425

**Published:** 2013-10-27

**Authors:** Faraidoon Haghdoost, Mohammad Mehdi Baradaran Mahdavi, Alireza Zandifar, Mohammad Hossein Sanei, Behzad Zolfaghari, Shaghayegh Haghjooy Javanmard

**Affiliations:** ^1^Medical Students' Research Center, Isfahan University of Medical Sciences, Isfahan 81745-319, Iran; ^2^Physiology Research Centre, Department of Physiology, Isfahan University of Medical Sciences, Isfahan 81745-319, Iran; ^3^Department of Pathology, Isfahan University of Medical Sceinces, Isfahan 81745-319, Iran; ^4^Department of Pharmacognosy and Isfahan Pharmaceutical Sciences Research Center, School of Pharmacy, Isfahan University of Medical Sciences, Hezar Jarib Avenue, Isfahan 81745-319, Iran

## Abstract

*Objectives*. The aim of the present study was to evaluate the effect of *Pistacia atlantica* resin extract on the rat skin burn wound healing. *Methods*. Thirty-two Wistar rats were divided into four groups and treated by vehicle, 5%, 10%, and 20% concentration of *Pistacia atlantica* resin extract for 14 days (G1, G2, G3, and G4, resp.). The efficacy of treatment was assessed based on reduction of burn wound size and histological and molecular characteristics. *Results*. **α**-Pinene (46.57%) was the main content of essential oil of resin. There were no statistically significant differences between groups according to wound size analysis. The mean histological wound healing scores were not statistically different. Capillary counts of G2 and G3 were significantly higher than those of the G1 (*P* = 0.042 and 0.032, resp.). NO concentration in wound fluids on the 5th day of study was not significantly different between groups (*P* = 0.468). But bFGF concentration in G2 and G3 and PDGF concentration in G3 were significantly higher in comparison to G1 (*P* = 0.043, 0.017, and 0.019, resp.). *Conclusion*. Our results revealed that *Pistacia atlantica* resin extract has a concentration-dependent effect on the healing of burn wounds after 14 days of treatment by increasing the concentration of bFGF and PDGF and also through improving the angiogenesis.

## 1. Introduction

Since ancient times, plants have played a major role in the treatment of many diseases, especially in the eastern countries. There are documents showing that Persians were pioneers in using plants for medical purposes. There are 7500–8000 plant species in Iran [1]. *Pistacia* (Persian name: Bane) is a genus of the family Anacardiaceae. Among 15 known species of pistachios, only some species grow in Iran, such as *Pistacia vera *L.,* Pistacia khinjuk *Stocks, and *Pistacia atlantica *Desf. These have played an important role in folk medicine and have been used in treatment of eczema, throat infection, renal stone, and asthma. They also act as astringent, anti-inflammatory, antipyretic, antibacterial, antiviral, pectoral, and stimulant [2].

*Pistacia atlantica* is a plant native to a number of countries such as Iran, Iraq, and Turkey. The oleoresin of this plant is used for making chewing gum in Iran and has also been used traditionally in the treatment of peptic ulcer disease and as a mouth freshener [3]. This plant's extract is also used traditionally as a wound dressing in Kurdistan of Iran [4].

Millions of people suffer from burn-related disabilities and disfigurements which impose psychological, social, and economic burdens on both burn survivors and their families. In 2002, 330,000 deaths were estimated, directly or indirectly, related to burn injury [5, 6]. Burn wound healing is a critical component of the burn patients' successful recovery that is associated with large impact on health care costs [7, 8].

Wound healing is a well-ordered response to injury starting with inflammation dominated early phase, progressing to the repair and remodeling of wound tissue. There are three phases in the wound healing process: inflammation, proliferation, and remodeling [9]. The inflammatory phase involves release of cytokines and growth factors, influx of neutrophils and macrophages and creation of an initial matrix [10]. The proliferation phase is characterized by angiogenesis, collagen deposition, reepithelialization, and wound contraction. Endothelial cells initiate angiogenesis and fibroblasts exert collagen and fibronectin to form new extracellular matrix in granulation tissue [11, 12]. The final phase is matrix remodeling that is characterized through collagen deposition by fibroblasts and formation of an organized network [13]. The complex process of wound healing is regulated by signaling network that involves numerous cytokines, chemokines, and growth factors such as platelet derived growth factor (PDGF) and fibroblast growth factor (FGF) that have been reported to accelerate various aspects of wound healing [14, 15].

PDGF is a potent mitogen for all mesenchymal cells and acts as a chemoattractant for neutrophils, monocytes, and fibroblasts. It also stimulates synthesis of fibronectin, glycosaminoglycan, and collagenase [15–18]. Fibroblast growth factors (FGFs) are a family of structurally related polypeptides which are mitogenic for an extensive range of cell types. Basic fibroblast growth factor (bFGF) is a growth factor of FGF family which induces DNA synthesis and angiogenesis, stimulates extracellular matrix formation, and down-regulates collagen type one synthesis [17, 19].

In normal body condition, there is a balance between free radicals and natural scavengers. But during the traumatic state the balance is lost and reactive oxygen species (ROSs) are superior in number. Burn trauma not only up-regulates free radical production but also impairs antioxidant defense mechanism, rendering burn patients more susceptible to ROS-mediated injury through cellular DNA and protein damage [20, 21]. Nitric oxide (NO) is an intercellular signaling molecule that the efficiently balanced production of it plays an important role in burn healing. The highly valuable effect of bioavailable NO is ascribed to scavenging of superoxide, as the major component of oxidative stress. NO has also beneficial effect on angiogenesis, inflammation, matrix deposition, and remodeling [22, 23].

To the best of our knowledge, there is no previous report on wound healing properties of *Pistacia atlantica* resin extract. The objective of our study was to examine wound healing potential of *Pistacia atlantica* resin extract.

## 2. Material and Method

### 2.1. Animals and Experimental Protocol

Thirty-two female Wistar rats weighing 250 ± 20 g at the burn time, from the Razi Institute of Iran, were housed in the animal unit (12-hour light/dark cycle, temperature approximately 23°C) at least two weeks prior to the experiments. The rats were housed in individual cages with free access to water and food pellets. The rats were randomly divided into four groups and each rat got a number to perform the blindness of analyzers. After creating burn wounds, each group was dressed by a different extract dose of *Pistacia atlantica* resin for 14 days. At the 14th day, the animals were sacrificed and the wounds were separated to determine the healing grade by microscopic evaluation.

### 2.2. Burn Injury

The rats were anesthetized with intra-peritoneal injection of ketamine (50 mg/kg) and xylazine (5 mg/kg). The dorsa of the animals were shaved and burn injury was induced by applying an aluminum plaque (1.5 ∗ 1.5 cm) on the skin of the rats for 10 seconds which was heated to 100 degree centigrades in a dry oven, to create a deep dermal burn wound. All the procedure was done by the same person to minimize the bias of differences in the force the person applies. The reliability of this method in the production of full thickness burns has previously been validated by Koizumi et al. [24].

### 2.3. Plant Collection and Extract Preparation

The resin of *Pistacia atlantica *was collected from Zagros Mountains in Kurdistan province in October 2010. The plant was identified by Prof. M. R. Rahiminezhad, Herbarium Department of Biology, Faculty of Sciences, Isfahan, Iran, and voucher specimen (no. 2226) is deposited at the Department of Pharmacognosy, Isfahan University of Medical Sciences.

For quality control, *Pistacia atlantica* resin (50 g) was hydrodistilled (with 1.2 L water) in a clevenger-type apparatus for 4 hours according to British Pharmacopoeia guideline [25]. Pale yellow oil from the resin was obtained (12% v/w). One gr Carbopol 934 was added to 100 mL deionised water and was mixed and then triethanolamine was added drop by drop to the solution to obtain a desirable gel that would be used as a vehicle. To prepare the different concentrations of the ointment (resin extract), we added 5, 10, and 20 grams of the resin to 95, 90, and 80 grams of the vehicle to obtain 5%, 10%, and 20% concentration ointment, respectively. The control group was treated only by the vehicle. 

### 2.4. GC-MS Analysis

GC-MS analysis was performed on a Hewlett Packard 5792A mass selective detector coupled with a Hewlett Packard 6890 gas chromatograph, equipped with a HP-5MS capillary column (30 m × 0.25 mm, film thickness 0.25 *μ*m). The GC operating conditions were as follows: carrier gas, helium with a flow rate of 2 mL/min; column temperature, 60–280°C at 4°C/min; injector and detector temperatures, 280°C; volume injected, 0.1 mL of the oil; and split ratio, 1 : 50. The MS operating parameters were as follows: ionization potential, 70 eV; ion source temperature, 250°C; resolution, 1000; ionization current, 750 *μ*A; and mass range, 35–425.

Identification of the constituents was based on computer matching against the library spectra (Library Database Wiley 275L), their retention indices with reference to an n-alkane series in a temperature programmed run, interpreting their fragmentation pattern, and comparison of the mass spectra with those reported in the literature [26].

### 2.5. Treatment

The animals were divided into four groups. From the first day, wounds of the rats were dressed by 20 mg of the *Pistacia atlantica* extract for each group daily. Group one (G1) was the control group in which the burn wounds were covered by an ointment base (vehicle) without any extract. Group 2 (G2), Group 3 (G3), and Group 4 (G4) received daily application of 5%, 10%, and 20% prepared extracts, respectively. 

### 2.6. Wound Size Assessment

**Figure 1:**
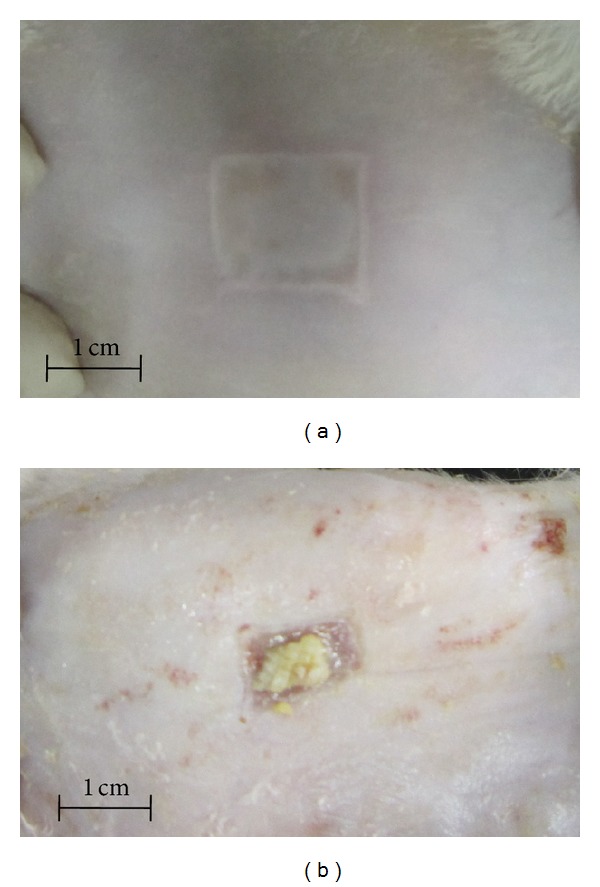
Macroscopic morphology of skin burn wound created by 10-second application of 1.5 ∗ 1.5 aluminum plaque which was heated to 100 degree centigrade. (a) Macroscopic morphology of the wounds after 14-day treatment by *Pistacia atlantica* resin extract (b).

The burn wounds were photographed after creating wound (first day) and at day 14, by the same instrument (Canon IXUS 200 IS Digital Camera) and settings, with fixed distance of camera from the wound and the same position of rats when imaging (Figure 1). Then the photos were analyzed by MATLAB R2009a software. Data from MATLAB software were pixels of wound image per pixels of one cm^2^. Differences between the wound size at the first day and also 14th day between groups and changes from the first day to 14th day were compared between groups. Also wound contraction (=100 − [(wound size on 14th day/wound size on 1st day) ∗ 100]) was assessed [27].

### 2.7. Histological Assessment

After the 14th day, all the rats were scarified and the wounds were separated. All wound tissue specimens were fixed in 10% neutral-buffered formalin for at least 24 h at room temperature. After fixation, vertical sections to the anterior-posterior axis of the wound were dehydrated in graded ethanol, cleared in xylene, and embedded in paraffin. Four-micron-thick sections were mounted on glass slides, dewaxed, rehydrated to distilled water, and stained with hematoxylin and eosin. For histological evaluation, all slides were examined by two pathologists, without knowledge of the prior treatment, under a microscope from ×20 to ×100 magnifications. The histological score adopted in this study was performed according to the previous study concerning wound healing in experimental models. The criteria used as histological scores of wound healing are summarized in Table 1 [28]. Also the slides were examined to count the capillary count (capillary density). The presence of a capillary was defined according to the following criteria: (1) a lumen, (2) red blood cells and (3) an endothelial cell lining the lumen. The capillary counting for each slide was done in the ×400 magnificent view in 4 different regions and the mean was reported [29]. 

### 2.8. Determination of bFGF, PDGF, and NO in Wounds Fluids

Three samples of wound fluid were collected using sterile nitrate-free absorbent paper strips placed on the edges of the wound for 10 min, in order to measure bFGF, PDGF, and NO on the 5th day of the study. This method for the measurement has been validated for other sample types, particularly for tears [30–32]. For bFGF and PDGF measurement, protein elution from the Schirmer strips was performed by stirring the strips in 0.5 mL of buffer (50 mM Tris, 50 mM NaCl, 0.05% Brij 35, pH 7.6) for at least 2 h at +4°C. For wound fluid NO determinations, filter paper was placed in 0.5 mL of distilled water [33]. The amount of bFGF and PDGF in wounds fluid was measured using enzyme-linked immunosorbent assay by available reagents and recombinant standards (R&D Systems, Minneapolis, MN) according to the manufacturer's instruction in 5th day samples. The total NO level of wound fluid was measured using the Griess assay after conversion of NO_3_ to NO_2_ with the NO_3_ reductase enzyme as described previously [34].

### 2.9. Statistical Analysis

All data are expressed as the mean ± the standard deviation (mean ± SD). A statistical software package, SPSS (version 16), was used to perform statistical analysis. The data were tested for normality and homogeneity of variance. Data were analyzed by analysis of variance (ANOVA), followed by a post hoc multiple comparison. For the histological results, statistical analysis was performed using Kruskal-Wallis test. Statistical significance was accepted at *P* < 0.05.

## 3. Results

The *Pistacia atlantica* resin composition was identified and is reported in Table 2. *alpha*-Pinene (46.57%) was the main constituent followed by *beta*-pinene (9.08%), *trans*-verbenol (6.41%), sabinene (4.49%), and *trans*-pinocarveol (4.05%). 

At the first day of experiment, there was no significant difference in the mean weight of groups (mean weight of all the animals was 194.37 ± 19.37 and the *P* value of comparing the groups was calculated as 0.198). As it is shown in Table 3, there was no significant difference in the wound size in the first day and also 14th day. The wound size decreases in the G1, G2, G3, and G4 were 0.98 ± 0.35, 1.43 ± 0.39, 1.30 ± 0.40 and 1.09 ± 0.34, respectively and the *P* value calculated for comparing differences was 0.103. Wound contraction for G1, G2, G3, and G4 was 41.98 ± 15.93, 58.17 ± 15.08, 46.52 ± 16.03 and 43.50 ± 10.23 percent, respectively (*P* = 0.091).

**Figure 2:**
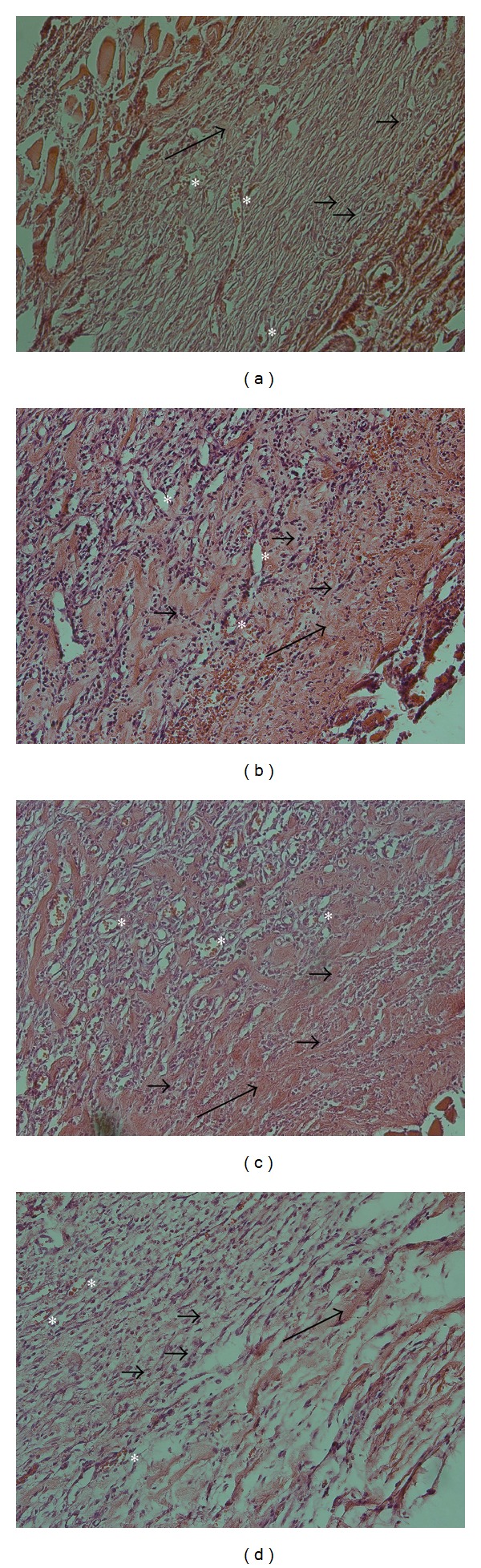
Histopathology of burn wounds at day 14 stained with H&E (200x). (a) Control group. (b) Group treated by 5% extract concentration. (c) Group treated by 10% extract concentration. (d) Group treated by 20% extract concentration. Stars show capillaries. Small arrows show fibroblasts. Long arrows show granulation.

Table 4 shows that there was no significant difference between groups in the rate of wound healing score determined by microscopic analysis of wounds. The capillary count analysis showed that there is a significant difference between groups and G2 and G3 had a higher capillary count (Table 4). G4 had a lower capillary count than G2 and G3 but it was not statistically significant (0.753 and 0.680, resp.).  Figure 2 is showing the histopathology of the wounds in different groups of the study after 14 days.

The concentration of NO (*μ*mol/mL) (mean ± standard error) in the burning wound fluids of G1, G2, G3, and G4 was 3.20 ± 0.97, 2.92 ± 0.95, 5.18 ± 0.61, and 3.68 ± 0.69, respectively. The differences were not statistically significant (*P* = 0.232).

The concentration of bFGF and PDGF in the wound fluids is shown in Table 5. There is a significant difference in the level of PDGF concentration in the wound fluids between groups (*P* = 0.034). Also differences between groups are statistically significant in the case of bFGF (*P* = 0.007). Differences between G1 and the two groups of G2 and G3 are statistically significant (*P* value of 0.043 and 0.017, resp.).

## 4. Discussion

The complex process of wound healing is regulated by an equally complex signaling network involving numerous growth factors, cytokines, and chemokines [14, 15]. The aim of our study was to investigate the effect of *Pistacia atlantica* resin extract on burning wound healing because of its wide traditional use in Kurdistan of Iran to cure wounds especially burning wounds. We evaluate morphological, histopathological, and biochemical parameters for wound healing potential assessment of *Pistacia atlantica* resin extracts in burned rats.

In this study, the wound size analysis results showed that although there were no significant differences between groups but in all groups treated by resin extract, decreasing in wound sizes was more than G1. Also wound contraction analysis had the same results and showed that although there is no significant difference between groups, all groups had higher percentages of contraction than the G1. Wound healing scoring showed that the healing rates of groups treated by resin extract (G2, G3 and G4) were higher than G1 but statistically not significant. Since nearly all groups got a score lower than 50% of our scoring system, conducting similar studies in a study time more than 14 days and also using larger sample sizes are recommended. It is reported that myofibroblasts are cells specialized in wound contraction and synthesis of new extracellular matrix. Normal wound myofibroblasts contribute to angiogenesis during wound healing that is mediated by increase in tissue inhibitor of metalloproteinase [35].

Our results showed that capillary count in groups treated with *Pistacia atlantica* extract is higher than that of the G1 and differences of G1 with G2 and G3 were statistically significant. Although differences of G4 with G2 and G3 were not statistically significant, the capillary count for G4 was lower than them. Our results showed that higher doses of *Pistacia atlantica *resin extract are associated with lower angiogenesis and this is consistent with other studies that revealed that mastic oil from *Pistacia lentiscus *has a dose-dependent effect on vascular endothelial growth factor (VEGF) concentration and also angiogenesis. Loutrari et al. showed that in higher doses of mastic oil extraction from* Pistacia lentiscus*, angiogenesis will reduce. Their results showed that VEGF concentration in groups treated by low dose of extract is higher than in the group using vehicle without any extract. But in higher doses, VEGF is lower even than the group treated by vehicle [36]. Djerrou et al. revealed that *Pistacia lentiscus *virgin fatty oil significantly promotes wound contraction and reduces epithelialization period in rabbit model [37]. Another study showed that *Pistacia lentiscus* fatty oil improves the burn wound healing properties of honey when mixed in rabbit model [38]. 

Analysis of NO, bFGF and PDGF concentration in wound fluids on the 5th day of study showed that the concentration of NO in wound fluid was not significantly different between groups. G1 had lower PDGF concentration than all other groups and the difference with G3 was statistically significant. The bFGF concentration in groups treated by low dose of extract was higher than control group and the differences with G2 and G3 were statistically significant. The burnt wound healing is a complex process and requires a well-coordinated collaboration of different tissues and cells. Angiogenesis has an important role in the healing process of skin burns. The angiogenesis starts fast and about three days after producing the burn, endothelial precursor cells will be identified. The density of the vessels will grow about two weeks. After that, they reduce progressively while the tissue of granulation become mature [39]. This may be the explanation for our results that showed significant differences between groups when considering capillary count, PDGF, and bFGF but no significant differences when we compared wound healing scores and wound sizes. As we mentioned previously, healing score that all groups received after 14 days was nearly lower than 50% of the total score and conducting studies with duration of more than 14 days is recommended.

It has been shown that reducing NO production by NO synthase knockout mice impairs wound healing [40]. Furthermore, NO has a regulatory role in vascular endothelial growth factor (VEGF) through wound healing process. VEGF is a key angiogenic molecule with an important role in vascular permeability which implies the importance of VEGF in wound healing [41]. Also NO is effective in proliferations of vascular smooth muscle cells by PDGF as a potent smooth muscle chemoattractant and mitogen [42, 43]. bFGF is an important inducer of angiogenesis, in in vitro angiogenesis models of endothelial cells. bFGF could induce angiogenesis, and this action was linked to VEGF production through the activation of endothelial nitric oxide synthase in endothelial cells [44].

The burn wound represents a susceptible site for colonization of organisms with endogenous and exogenous origin [45]. Some studies showed that *Pistacia atlantica* extracts have considerable antimicrobial activity, specifically antifungal effect, and also are effective in reducing and scavenging the superoxide anions in vitro [2, 46]. An in vitro study evaluation of the biological activity of *Cedrus libani* (Pinaceae)—in which the main constituents of the cones ethanol extract are *α*-pinene (51.0%) and *β*-myrcene (13.0%)—against Herpes simplex virus type 1 (HSV-1) showed an interesting antiviral activity [47]. Some studies have reported an antibacterial effect for *β*-pinene and *α*-pinene. It has been proved that *α*-pinene has an interesting antibacterial effect [2, 48]. Our results showed that the main constituent of *Pistacia atlantica* was *α*-pinene (46.57%), so its antibacterial effect can be another reason of wound healing effect of this plant. 

## 5. Conclusion

Our results showed that *Pistacia atlantica *resin may be useful in the treatment of burning wounds by increasing the concentration of bFGF and PDGF and also by increasing the angiogenesis. Plants are not only cheap but also safe, so they can be used widely to treat wounds. Interestingly our results showed that the effect of *Pistacia atlantica* resin on burning wound healing (after 14 days of treatment by the plant) is dose-dependent and in higher doses has reverse effect and lower healing will occur. We suggest that repeating the same study with larger sample size, longer period of time (more than 14 days), and also more divided doses will be more informative. 

## Figures and Tables

**Table 1:** Criteria to evaluate histological scores of wound healing for H&E staining.

Score	Criteria
1–3	None to minimal cell accumulation. No granulation tissue or epithelial travel.

4–6	Thin, immature granulation tissue that is dominated by inflammatory cells but has few fibroblasts, capillaries, or collagen deposition. Minimal epithelial migration.

7–9	Moderately thick granulation tissue can range from being dominated by inflammatory cells to more fibroblasts and collagen deposition. Extensive neovascularization. Epithelium can range from minimal to moderate migration.

9–12	Thick, vascular granulation tissue dominated by fibroblasts and extensive collagen deposition. Epithelium partially to completely covering the wound.

**Table 2:** Composition of *pistacia atlantica* resin oil.

No.	Compound	RI*	%**
1	Tetramethylcyclopentene	839	0.23
2	*alpha*-Pinene	942	46.58
3	Camphene	964	2.03
4	Verbenene	967	1.73
5	Sabinene	982	4.49
6	*beta*-Pinene	988	9.08
7	Myrcene	995	0.19
8	*alpha*-Phellandrene	1009	0.49
9	*delta*-3-Carene	1031	1.58
10	*alpha*-Terpinene	1017	0.28
11	Methane	1026	0.20
12	Para cymene	1025	1.49
13	Limonene	1029	3.40
14	Cineole	1031	0.38
15	*trans*-*beta*-Ocimene	1042	1.08
16	*gamma*-Terpinene	1060	0.53
17	*cis*-Sabinene hydrate	1070	0.38
18	Linalool	1097	1.33
19	*alpha*-Campholene	1128	1.28
20	*trans*-Pinocarveol	1143	4.05
21	*cis*-Verbenol	1145	1.14
22	*trans*-Verbenol	1151	6.41
23	Pinocarvone	1164	0.30
24	Terpineol-4	1179	1.01
25	*alpha*-Terpineol	1191	0.78
26	Myrtenol	1196	1.73
27	*trans*-Carveol	1219	0.57
28	Chrysanthemyl acetate	1261	0.23
29	Bornyl acetate	1284	0.38
30	*alpha*-Terpinyl acetate	1348	0.32

*Retention indices on HP-5MS capillary column.

**Calculated from TIC data.

**Table 3:** Wound size (cm_2_) analysis of the studied group in first and 14th days of the study.

	First day	14th day	Wound contraction^*∧*^
	mean ± SD (*n*)	mean ± SD (*n*)	mean ± SD (*n*)
Control group	2.36 ± 0.18 (8)	1.37 ± 0.41 (8)	41.98 ± 15.93 (8)
Group treated by 5% EC	2.48 ± 0.22 (8)	1.05 ± 0.48 (8)	58.17 ± 15.08 (8)
Group treated by 10% EC	2.30 ± 0.31 (8)	1.00 ± 0.43 (7)	46.52 ± 16.03 (7)
Group treated by 20% EC	2.47 ± 0.24 (8)	1.38 ± 0.15 (8)	43.50 ± 10.23 (8)
^†^ *P* value	0.389	0.133	0.091

EC: extract concentration. ^*∧*^Data are given as percentages, ^†^by comparison of all groups (ANOVA test). *n*: number of animals.

**Table 4:** Wound healing score and capillary count analysis of studied groups after H&E staining.

Groups	Wound healing score	Capillary count	*P* value^†^
Control group	3.28 ± 0.28	09.28 ± 1.01	
Group treated by 5% EC	4.20 ± 0.58	15.12 ± 1.74	0.042*
Group treated by 10% EC	6.40 ± 1.77	15.37 ± 1.23	0.032*
Group treated by 20% EC	3.40 ± 0.42	13.12 ± 1.55	0.274
^††^ *P* value	0.066	0.026**	

EC: extract concentration. Data are given as mean ± standard error, ^†^comparison of G1 with other groups for capillary count analysis, *significantly different from the control group, **statistically significant difference, ^††^by comparison of all groups (Kruskal-Wallis test).

**Table 5:** Evaluation and analysis of bFGF, PDGF and NO concentrations in the wounds fluid in 5th day of the study.

Groups	bFGF^*∧*^	*P* value^†^	PDGF^*∧*^	*P* value^†^	NO^*∧∧*^
	mean ± SD (*n*)		mean ± SD (*n*)		mean ± SD (*n*)
Control group	50.60 ± 0.43 (7)		18.64 ± 1.70 (6)		3.20 ± 0.97 (8)
Group treated by 5% EC	52.60 ± 1.71 (8)	0.043*	24.58 ± 7.05 (8)	0.255	2.92 ± 0.95 (7)
Group treated by 10% EC	52.90 ± 1.79 (8)	0.017*	28.89 ± 4.78 (7)	0.019*	5.18 ± 0.61 (8)
Group treated by 20% EC	51.17 ± 1.00 (8)	0.589	24.70 ± 6.96 (8)	0.240	3.68 ± 0.69 (8)
^††^ *P* value	0.007**		0.034**		0.232

EC: extract concentration. Data is given as mean ± SD, ^†^comparison of G1 with other groups, ^*∧*^concentration (pg/mL), ^*∧∧*^concentration (*µ*mol/mL), *significantly different from the control group, **statistically significant difference, ^††^by comparison of all groups (ANOVA test). *n*: number of animals.
